# The effect of POSS nanoparticles on crosslinking of styrene-butadiene rubber nanocomposites

**DOI:** 10.55730/1300-0527.3548

**Published:** 2023-02-07

**Authors:** Seda BEKİN AÇAR, Mehmet Atilla TAŞDELEN, Bağdagül KARAAĞAÇ

**Affiliations:** 1 Department of Polymer Materials Engineering, Faculty of Engineering, Yalova University, Yalova, Turkey; 2 Department of Chemical Engineering, Faculty of Engineering, Kocaeli University, Kocaeli, Turkey

**Keywords:** Nanocomposites, nanoparticles, polyhedral oligomeric silsesquioxane, rubber, styrene-butadiene rubber

## Abstract

The effect of octaisobutyl-polyhedral oligomeric silsesquioxane (OIB-POSS) as a nanosized reinforcement on the cure kinetics, crosslinking density, and mechanical properties of styrene-butadiene rubber (SBR) nanocomposites was examined in this study. For this purpose, SBR compounds with various OIB-POSS nanoparticle loadings at 1, 3, and 5 phr were prepared and their results were compared with a reference compound without OIB-POSS. When 1 phr of OIB-POSS was added to the rubber matrix, the elongation at break values and tensile strength of the corresponding nanocomposite increased by 24.1% and 29.2% compared to the reference sample, respectively. The presence of OIB-POSS nanoparticles and their random distribution in the SBR matrix was confirmed by transmission electron microscopy. The crosslinking density of nanocomposites was calculated by the Flory-Rehner method and a decrease was observed with the addition of OIB-POSS nanoparticles. In addition, thermal aging process as 70 °C for 70 h was applied to vulcanized samples. It was noted that the mechanical properties of SBR/OIB-POSS nanocomposites remarkably improved, whereas their crosslinking densities gradually decreased after thermal aging.

## 1. Introduction

Rubber composites are generally prepared with various reinforcements having different particle size and surface energy according to the application areas of rubbers [1]. The reinforcement-rubber interaction is an important parameter for the improving of physical properties of corresponding composites. Most of these reinforcements are minerals or carbonized organic compounds in micron size. By reducing the size of the reinforcements from micrometers to nanometers, the nanocomposites reinforced with nano-sized particles exhibit superior mechanical, thermal and barrier, and permeability properties [2]. Recently, the development of rubber-based nanocomposites has attracted great interest and nano-sized fillers such as layered silicates, carbon nanotubes, metal oxides and polyhedral oligomeric silsesquioxane (POSS) nanoparticles have been widely used to prepare them [3].

Styrene-butadiene rubber (SBR) is one of the synthetic nonpolar rubber types and synthesized from styrene and 1,3-butadiene monomers [4]. Besides, SBR has been used as a raw material in various industries; it is mainly used in the tire industry due to its valuable properties such as good weather resistance, humidity, incision impedance, and high filler loading capacity [5–8]. To improve the SBR properties, spherical carbon black and silica have long been used as reinforcing fillers at high loadings. However, excess loading of fillers causes agglomeration in rubber matrices, which has a negative effect on properties including reduced fatigue performance of rubber composites [9]. Therefore, in recent years, nano-sized fillers have attracted more attention in academic and industrial fields. They provide significant improvement in the mechanical properties even at low filler concentration because of their high aspect ratio and enhanced interface compatibility [10].

The POSS is a new-generation three-dimensional nanofiller having a silica-like core surrounded by a shell of organic groups and widely used in polymers as it provides reinforcement and stabilizing properties to polymer materials [11–13]. The hybrid POSS is a reinforcing nanofiller suitable for elastomeric composites, combining the most beneficial properties of both organic and inorganic systems [14]. POSS nanoparticles can be effectively combined with polymer and rubber matrices by either physical blending or chemical bonding because of their organic/inorganic hybrid natures [15, 16]. POSS nanoparticles improve the mechanical, thermal, and flame retardant properties of polymer and rubber materials [13, 17].

In this study, it was aimed to use OIB-POSS as a potential reinforcing agent for SBR, unlike our previous study, in which POSS nanoparticles containing reactive groups were chemically bonded to the SBR matrix and involved in the vulcanization mechanism. The effect of OIB-POSS nanoparticles added to the SBR matrix as nonreactive reinforcement nanofillers at 1, 3, and 5 phr on the rubber compounds was investigated through crosslinking, mechanical, physical, and rheological properties. The change in the properties of SBR nanocomposites after the thermal aging process was also examined.

## 2. Materials and methods

### 2.1. Materials

**Figure 1:**
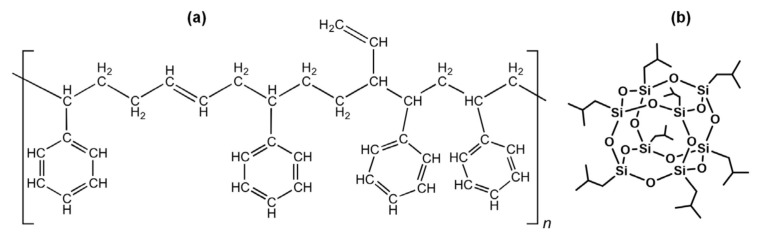
The chemical structures of SBR (a) and OIB-POSS (b).

Styrene-butadiene rubber (having 27% styrene content, SBR 1502) was supplied by Arlanxeo, Germany. Nonfunctional octaisobutyl POSS (OIB-POSS, molecular weight of 870.60 g/mol) was obtained from Hybrid Plastics Inc., USA and used as received without purification. The chemical structures of SBR and OIB-POSS are given in Figure 1. All other ingredients including *N*-cyclohexyl-2-benzothiazole sulfenamide (CBS), N-Isopropyl-N′-phenyl-1,4-phenylenediamine (IPPD, ozon wax, stearic acid (SA), sulfur (S), tetramethylthiuram disulfide (TMTD), 2,2,4-trimethyl-1,2-dihydroquinoline (TMQ), and zinc oxide (ZnO) in rubber recipes were obtained from Rubber Chem, Turkey and used as received.

### 2.2. Preparation of SBR/OIB-POSS nanocomposites

A laboratory type banbury was used for the preparation of SBR/OIB-POSS nanocomposites according to the procedure of our previous study [5]. The details of formulation are listed in Table 1. First, SBR was masticated in banbury for 2 min to prepare rubber nanocomposites. Subsequently, OIB-POSS nanofiller selected to be 1, 3, or 5 phr was added to the masticated SBR and compounded in the same mixer for 1 min. This compound was mixed with ZnO as activator, and SA as lubricant for 0.5 min. Then, mixing of stabilizers (TMQ, IPPD, and ozone wax) was carried out for another 0.5 min. Finally, the remaining components (CBS, S, and TMTD) were included into the compound and mixed for another 1 min. Overall, all SBR/OIB-POSS compounds were obtained after 5 min of total mixing process at about 80 °C.

The SBR/OIB-POSS compounds were successively vulcanized using a hydraulic hot press at 160 °C. During the vulcanization, their optimum cure times were determined by a moving die rheometer (MDR). Test specimens were obtained by cutting in accordance with the required dimensions and standards of tests to be applied to the compounds. Rubber nanocomposites were kept in an air-circulating oven for 70 h at 70 °C to investigate the effect of thermal aging.

### 2.3. Characterization

The rheometer parameters of the SBR/OIB-POSS nanocomposites and the reference compound were determined by a moving die rheometer (MDR, Alpha Technologies) in accordance with ASTM D5289 (2019). The chemical structures of SBR/OIB-POSS nanocomposites were characterized by ATR technique using Spectrum Two, Perkin-Elmer (USA) Fourier-Transform Infrared Spectrum (FT-IR) equipment at room temperature. Mechanical properties of reference sample and rubber nanocomposites were determined with an Instron Universal Tester (Model 3345) in accordance with ASTM D412 (2019). The speed of the crosshead was applied to be 500 mm/min. Zwick Shore A type durometer in accordance with ASTM D2240 (2015) was used to test the hardness of vulcanized nanocomposites. Also, compressions set tests of samples were performed properly with ASTM D395 (2018). To examine the dispersion of OIB-POSS nanoparticles in the rubber matrix and the morphology of SBR/OIB-POSS nanocomposites, transmission electron microscopy (TEM) analysis was performed with the instrument Joel JEM-2100 (UHR) Gatan, (USA) at 300 kV.

Crosslinking densities of cured and aged SBR/OIB-POSS nanocomposites were determined by equilibrium solvent-swelling ratios in toluene, densities of the rubber matrix and toluene [18], volume fractions of the polymer and solvent [19], and polymer-solvent interaction parameter [19–21] according to Flory-Rehner equation.

## 3. Results and discussion

In this study, influence of nonfunctionalized OIB-POSS nanoparticles on the rheological, physical, mechanical, morphological, and crosslinking properties SBR nanocomposites were investigated. For this purpose, the OIB-POSS nanofiller at 1, 3, and 5 phr was intensively mixed with SBR and then were vulcanized at 160 °C. The effect of thermal aging (70 hours at 70 °C) on SBR/OIB-POSS nanocomposites was also investigated.

### 3.1. Rheological characteristics

Rheometer data with important parameters of the reference compound and rubber nanocomposites were determined at 160 °C. The average results of rheometer tests repeated three times are presented in Table 2. The minimum torque (ML) values were proportional to the composition viscosity.

The ML values of the SBR nanocomposites and the reference sample were quite similar; however, the maximum torque (MH) values were remarkably decreased by increasing OIB-POSS loading. In addition, the cure extent (CE) value, which is related to the crosslinking degree of rubber and calculated from the difference between MH and ML values in the literature, decreased as the amount of OIB-POSS increased [22]. This may be because bulky POSS cages both increased the steric barrier between double bonds and sulfur atoms and decreased the crosslinking density in the rubber nanocomposites. Moreover, the optimum curing time (t_90_) values of SBR/OIB-POSS nanocomposites did not change significantly with the increase of OIB-POSS amount compared to the reference compound. The absence of a significant change in optimum curing time in the presence of OIB-POSS could be attributed to the fact that sulfur was not consumed in the early stage of vulcanization and there was no dominant reaction between OIB-POSS and sulfur.

One of the important parameters characterizing the vulcanization of rubber compounds is the cure rate index (CRI) and it was calculated with the given equation.


(equation 1) $$CRI=\frac{100}{t_{90}-t_{s2}}.$$

According to the rheometer test performed at 160 °C, the CRI values of SBR/OIB-POSS nanocomposites were significantly lower than those of the reference sample. Therefore, it could be concluded that the presence of OIB-POSS in the SBR matrix reduced the curing rate. The decrease in CRI values during the vulcanization reaction indicated to reduce the crosslink density in the rubber nanocomposites [8].

### 3.2. FT-IR characterization

**Figure 2:**
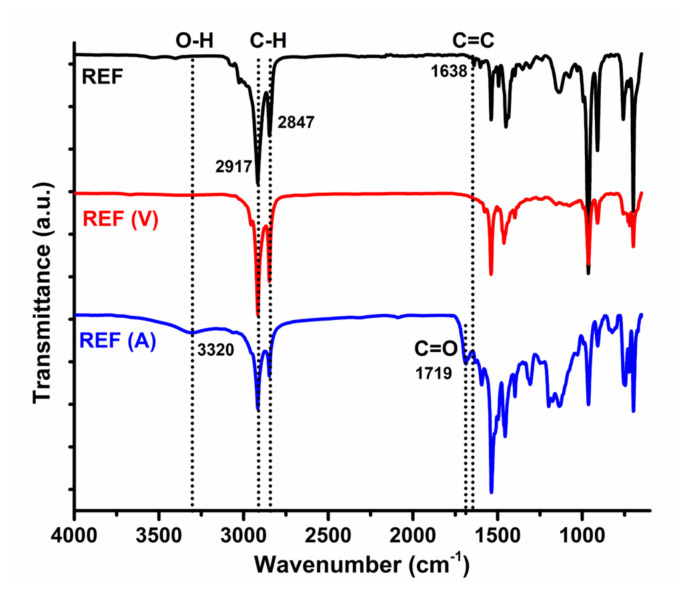
FT-IR spectra of the reference sample with nonvulcanized, vulcanized (V), and aged (A) forms.

**Figure 3:**
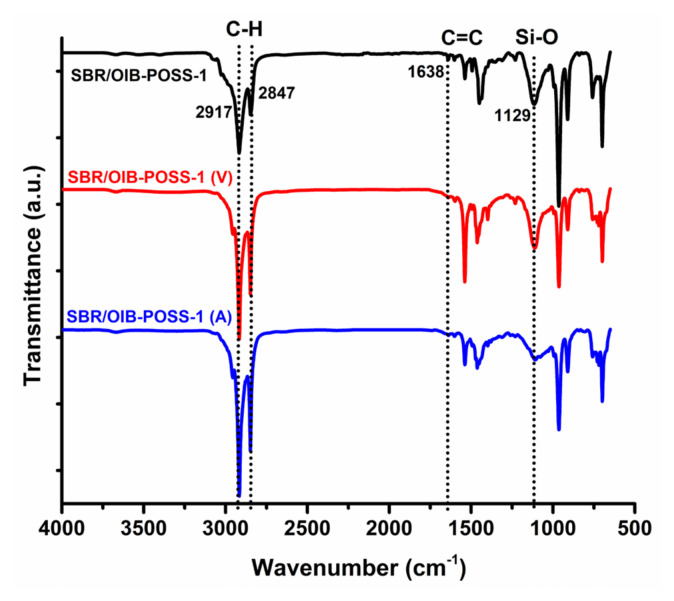
FT-IR spectra of the nonvulcanized, vulcanized, and aged SBR/OIB-POSS-1 nanocomposite samples.

The insertion of OIB-POSS into the SBR matrix was studied by FT-IR spectroscopy equipped with a diamond attenuated total reflection (ATR) instrument at room temperature. The FT-IR spectra of the reference and SBR/OIB-POSS-1 nanocomposite samples in nonvulcanized, vulcanized (V), and aged (A) steps are given in Figures 2 and 3 [7, 20, 23]. The complete disappearance of peak at 1638 cm^−1^ assigned to double bond of SBR matrix was the proof of the successful curing process (REF V). Moreover, the increase in peak intensity of bonds of hydroxyl and carbonyl groups in the aged reference compound spectrum (REF A) verified oxidative aging (Figure 2). Additionally, the stretching vibrations of aliphatic C-H peaks at 2917 cm^−1^ and 2847 cm^−1^ were evidently seen for SBR matrix. The bands at 1638 cm^−1^ and 968 cm^−1^ were attributed to the cis-CH= and trans-CH=CH- peaks of SBR, respectively. The aromatic substitution, another characteristic peak of SBR, was detected at 698 cm^−1^ [7, 24–26]. The presence of OIB-POSS in the SBR nanocomposites was confirmed by detecting the Si-O stretching band at 1129 cm^−1^ for the POSS groups in the FT-IR spectrum of the SBR/OIB-POSS-1 nanocomposite [27–29]. The successful vulcanization process was also verified by the absence of the peak at 1638 cm^−1^ in the FT-IR spectrum of vulcanized (SBR/OIB-POSS-1 (V)). In the literature, thermal oxidative aging of SBR results in the formation of oxygenated chemicals such as anhydrides, peresters, carboxylic acids, ethers, and alcohols [30, 31]. Therefore, the increase in hydroxyl and carbonyl bands of these products was the proof of the successful hydroxyl and carbonyl bonds. In our case, the FT-IR bands assigned the hydroxyl and carbonyl groups were clearly visible in the spectrum of the aged (SBR/OIB-POSS-1 (A)) sample.

### 3.3. Mechanical properties

**Figure 4:**
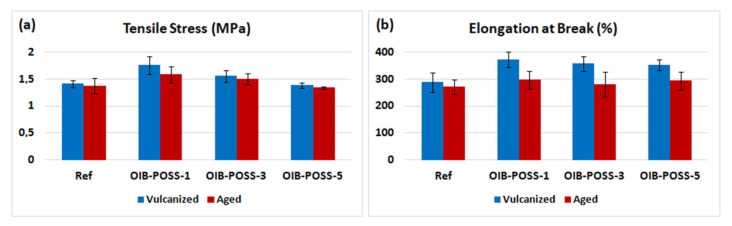
Tensile stress (a) and elongation at break (b) of the vulcanized and aged SBR/OIB-POSS nanocomposites

**Figure 5:**
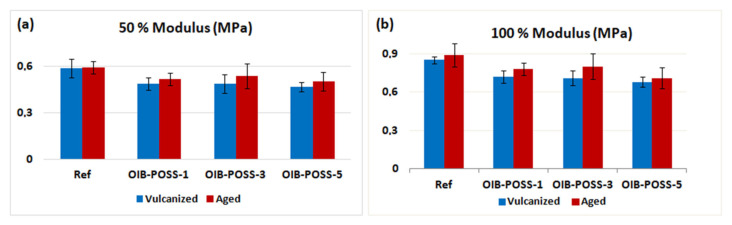
50% tensile modulus (a) and 100% tensile modulus (b) of the vulcanized and aged SBR/OIB-POSS nanocomposites.

The mechanical properties of SBR/OIB-POSS nanocomposites, in comparison with the conventional SBR reference compound, are shown in Figures 4 and 5 and summarized in Table 3. The average results of mechanical tests repeated five times are given with standard deviations. Among the prepared samples, the tensile strength of SBR/OIB-POSS-1 nanocomposite increased from 1.41 MPa to 1.75 MPa, with an improvement of 24.1% compared to the reference specimen. The dispersion or agglomeration of nanoparticles, interaction between SBR matrix and nanofillers, and particle distribution in cross-linked three-dimensional networks are of great importance in terms of mechanical strength of rubber nanocomposites [5]. Also, the tensile strength of the compounds is highly dependent on the amount and type of filler [4]. So, it was concluded that the good interaction between the nano-sized OIB-POSS reinforcement and the SBR matrix led to high mechanical strength. Compared to the reference sample, the elongation at break values increased by 29.2% when 1 phr OIB-POSS was added to the rubber matrix.

The elongation at break and tensile stress values of the obtained nanocomposites gradually decreased due to possible agglomerations and lower degree of crosslinking with the addition of more OIB-POSS, which could be supported by previous rheological data (Table 2). However, even in the SBR/OIB-POSS-5 nanocomposite, which was expected to exhibit the most possible agglomeration, both elongation at break and tensile stress values were greater than those of the reference sample. Tensile stress and elongation at break values of SBR/OIB-POSS nanocomposites decreased after thermal aging. It was also noted that the mechanical properties of aged SBR/OIB-POSS nanocomposite samples were significantly higher than the reference sample. On the other hand, the 50% and 100% tensile modulus values of the SBR/OIB-POSS nanocomposites were lower than the reference compound (Figure 4) due to the decrease in crosslinking density by addition of OIB-POSS into the rubber compounds. After thermal aging process, both 50% and 100% tensile modulus of aged SBR/OIB-POSS nanocomposites were greater than those of the vulcanized samples [30, 32–34].

The 50% and 100% tensile modulus values of the SBR/OIB-POSS nanocomposites were lower than the reference compound (Figure 5). The addition of OIB-POSS to the rubber compounds gave rise to a decrease in crosslinking density and modulus values. In addition, 50% and 100% modulus values of rubber nanocomposites increased due to increased stiffness after thermal aging [30, 32–34].

**Figure 6:**
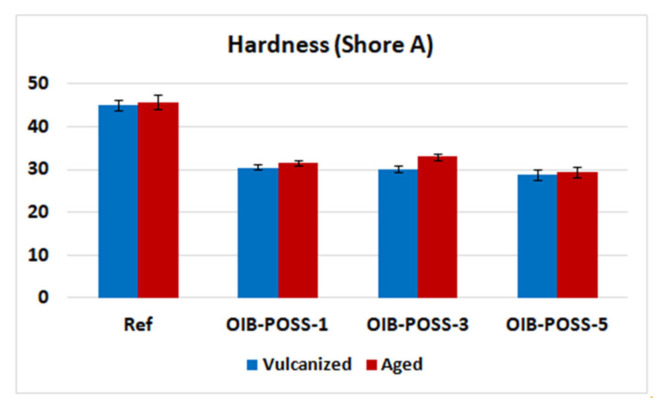
Hardness values of the vulcanized and aged SBR/OIBPOSS nanocomposites.

Shore A hardness and compression tests were performed according to ASTM D2240 (2015) and D395 method B (2018) to determine the effect of OIB-POSS nanoparticle loading on the resistance to indentation, permanent deformation of samples and to observe how the elastic properties change after prolonged compression at room temperature for 22 h and after thermal aging process at 70 °C for 70 h [35]. It was noted that the addition of OIB-POSS nanoparticles into the rubber compounds gave rise to a decrease in hardness values (Figure 6). Additionally, thermal aging process increased stiffness of the nanocomposite samples yielding the higher Shore A hardness values. It was also revealed that the permanent deformation value decreased when 1 phr of OIB-POSS was added to the reference sample but increased gradually with the addition of more OIB-POSS nanoparticles, which could be due to the change in crosslink density (Table 3) [36].

### 3.4. Crosslinking density

**Figure 7:**
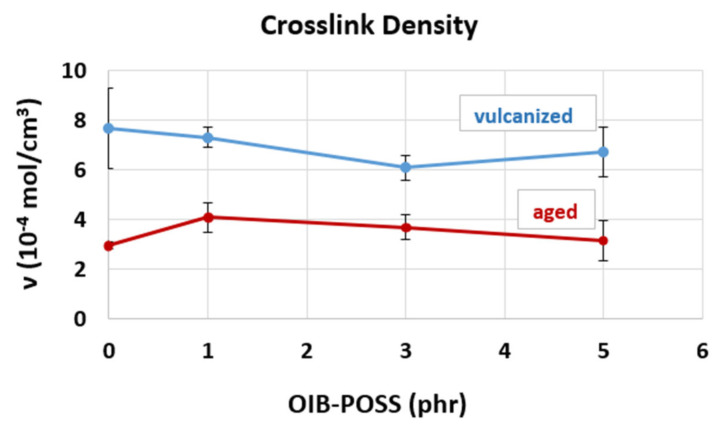
Crosslinking densities of vulcanized and aged refence and SBR/OIB-POSS nanocomposite samples.

The crosslinking densities (ν) of both vulcanized and aged SBR/OIB-POSS nanocomposites were determined by swelling measurements in toluene solvent using Flory-Rehner equation. The average results of the crosslink densities calculated from the three swelling tests are given in Figure 7 with standard deviations. It was determined that the crosslink densities of the nanocomposites decreased with increasing OIB-POSS concentration up to 3 phr. This could be attributed to possible agglomerations and inhomogeneous crosslinking points, which was also consistent with the rheological and mechanical tests data. During the thermal aging process of SBR, several complicated interactive reactions can take place, which causes the chain scission of SBR compound to soften and lose its elastic properties, while reducing the crosslink density. Therefore, the observed decrease in crosslink density of the samples after thermal aging of rubber nanocomposites could be attributed to potential chain breaking due to heat during the thermo-oxidative aging process [37, 38].

The reinforcement mechanisms in rubber nanocomposites are complex due to the multicomponent heterogeneous system and cross-linked structure. In addition to the interactions between filler-filler and filler-rubber, the particle size, specific area and dispersion level also affects the reinforcement of composites. Therefore, the decrease in crosslinking density cannot normally be explained by a single simple theory. Currently, the reinforcing mechanisms of the rubber nanocomposites have drawn significant interest with an emergence of novel fillers and various matrix/filler combinations [39].

### 3.5. Morphological properties

**Figure 8:**
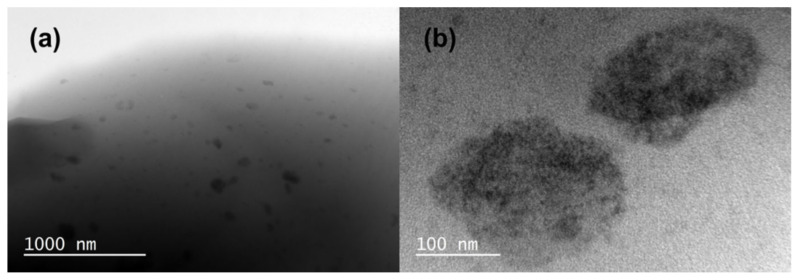
TEM micrographs of SBR/OIB-POSS-5 nanocomposite at low (a) and high (b) magnifications.

The morphological properties of the SBR/OIB-POSS nanocomposites were evaluated by TEM analysis from the rupture surface of the corresponding tensile sample. TEM images of the SBR/OIB-POSS-5 nanocomposite demonstrated fairly continuous and homogeneous distribution with some very rare OIB-POSS aggregates visible as black clusters between 50–200 nm (Figure 8-a). These dark clusters were intersections of the silicate layer bundles dispersed in styrene-butadiene rubber. Furthermore, at a higher magnification (Figure 8b), cubic OIB-POSS nanoparticles with diameters ranging from 2 to 5 nm, close in size to a single POSS molecules, were randomly distributed. Moreover, some aggregated OIB-POSS nanoparticles were still present in the corresponding rubber nanocomposite. In conclusion, the existence of OIB-POSS nanoparticles and their random distribution in the SBR matrix were confirmed by TEM analysis.

## 4. Conclusion

In this study, a series SBR/OIB-POSS nanocomposites with 1, 3, and 5 phr on the rubber compounds were prepared using a laboratory type banbury mixer, and their rheological, physical, mechanical, and crosslinking properties were investigated along with the reference SBR compound without nanofillers. The presence of OIB-POSS in the SBR nanocomposites was confirmed by detecting the Si-O stretching band at 1129 cm^−1^ for the POSS groups in the FT-IR spectrum of the SBR/OIB-POSS-1 nanocomposite. The successful vulcanization process was also verified by the absence of the peak at 1638 cm^−1^ in the FT-IR spectrum of vulcanized nanocomposite. As a result of the rheological test, it was determined that the addition of OIB-POSS to the rubber recipe reduced the CE value. The mechanical strength increased with the addition of 1 phr OIB-POSS to the SBR matrix, but further increase in the amount of POSS nanoparticle led to a negative effect on the mechanical properties due to possible agglomerations. The tensile strength of SBR/OIB-POSS-1 nanocomposite increased from 1.41 MPa to 1.75 MPa, with an improvement of 24.1% and the elongation of break values of corresponding nanocomposite increased by 29.2% compared to the reference sample, respectively. This increase in mechanical properties implied that the reinforcement effect was more dominant than the negative crosslinking effect. Accordingly, the crosslink densities of nanocomposites calculated by the Flory-Rehner method decreased with the addition of OIB-POSS, as predicted. TEM micrographs verified the presence and random dispersion of OIB-POSS in the SBR matrix. In addition, after the thermal aging process, the rigidity of the SBR/OIB-POSS nanocomposites increased while the mechanical strength decreased.

## Figures and Tables

**Table 1:** Recipes of SBR/OIB-POSS compounds.

Ingredients	Contents (phr)	Ingredients	Contents (phr)
Styrene-butadiene rubber (SBR)	100	N-isopropyl-n′-phenyl 1,4-phenylenediamine (IPPD)	1
Octaisobutyl POSS (OIB-POSS)	0/1/3/5	Ozon wax	1
Zinc oxide (ZnO)	5	Tetramethylthiuram disulfide (TMTD)	1
Stearic acid (SA)	2	N-cyclohexyl-2-benzothiazole sulfenamide (CBS)	1
2,2,4-trimethyl-1,2-dihydroquinoline (TMQ)	1	Sulfur (S)	1.5

**Table 2:** Rheological data of reference and SBR/OIB-POSS nanocomposite compounds.

Specimens	ML (dNm)	MH (dNm)	CE (dNm)	t_s2_ (min)	t_90_ (min)	CRI (min^−1^)
**Reference**	0.64	11.11	10.47	1.98	3.68	58.82
**SBR/OIB-POSS-1**	0.66	10.28	9.64	1.96	3.98	49.50
**SBR/OIB-POSS-3**	0.65	10.16	9.51	1.81	3.60	55.86
**SBR/OIB-POSS-5**	0.66	8.85	8.19	1.91	3.80	52.91

ML: Minimum torque

MH: Maximum torque

CE: Cure extent

t_s2_: Scorch time

t_90_: Optimum cure time

CRI: Cure rate index

**Table 3:** Mechanical properties of vulcanized and aged reference and SBR/OIB-POSS nanocomposite samples.

Samples	Tensile stress^b^ (MPa)	Elongation at break^b^ (%)	50% Modulus^b^ (MPa)	100% Modulus^b^ (MPa)	Hardness^c^ (Shore A)	Compression set^d^ (%)
**Reference**	1.41 ± 0.06	288 ± 35	0.59 ± 0.06	0.85 ± 0.03	45.0 ± 1.2	5.7 ± 0.8
**Reference (A)** a	1.37 ± 0.14	270 ± 27	0.59 ± 0.04	0.89 ± 0.09	45.8 ± 1.7	20.5 ± 2.1
**SBR/OIB-POSS-1**	1.75 ± 0.16	372 ± 28	0.49 ± 0.04	0.72 ± 0.05	30.5 ± 0.6	4.7 ± 0.2
**SBR/OIB -POSS-1 (A)** a	1.58 ± 0.15	297 ± 33	0.52 ± 0.04	0.78 ± 0.05	31.5 ± 0.6	26.8 ± 4.2
**SBR/OIB -POSS-3**	1.55 ± 0.11	357 ± 27	0.49 ± 0.06	0.71 ± 0.06	30.2 ± 0.8	4.8 ± 0.4
**SBR/OIB -POSS-3 (A)** a	1.50 ± 0.10	280 ± 45	0.57 ± 0.08	0.84 ± 0.10	33.0 ± 0.7	32.1 ± 4.9
**SBR/OIB -POSS-5**	1.38 ± 0.05	352 ± 20	0.47 ± 0.03	0.68 ± 0.04	28.8 ± 1.3	5.5 ± 1.0
**SBR/OIB -POSS-5 (A)** a	1.34 ± 0.02	294 ± 33	0.45 ± 0.06	0.71 ± 0.08	29.4 ± 1.1	32.9 ± 4.8

a (A): Aged samples

b According to ASTM D412 (2016)

c According to ASTM D2240 (2015)

d According to ASTM D395 Method B (2018)
